# An observational study of the effects of smoking cessation earlier on the clinical characteristics and course of acute exacerbations of chronic obstructive pulmonary disease

**DOI:** 10.1186/s12890-022-02187-5

**Published:** 2022-10-27

**Authors:** Xiaolong Li, Zhen Wu, Mingyue Xue, Wei Du

**Affiliations:** 1 Affiliated Haian Hospital of Nantong University, Haian, China; 2 Department of Pulmonary and Critical Care Medicine, Ruijin Hospital, Shanghai Jiao Tong University School of Medicine, 200025 Shanghai, China; 3 Institute of Respiratory Diseases, Shanghai Jiao Tong University School of Medicine, 200025 Shanghai, China; 4 Shanghai Key Laboratory of Emergency Prevention, Diagnosis and Treatment of Respiratory Infectious Diseases, 200025 Shanghai, China

**Keywords:** AECOPD, Smoking cessation, Therapeutics

## Abstract

**Background:**

Previous studies have focused on the negative effects of continued smoking on chronic obstructive pulmonary disease (COPD). However, few studies have investigated the positive effects of long-term smoking cessation on patients suffering from acute exacerbations of COPD.

**Methods:**

The study recruited and followed current or former smokers who had been hospitalized and diagnosed with AECOPD. An in-depth analysis of clinical and laboratory indicators was conducted.

**Results:**

125 patients were covered, including 72 short-term quitters and 53 long-term quitters. The results showed that long-term smoking cessation may result in milder dyspnea and cough, a higher oxygenation index, a lower arterial partial pressure of carbon dioxide, and milder pulmonary hypertension, airflow restriction, and gas retention in patients with AECOPD. However, despite the lower treatment intensity for long-term quitters, improvement in dyspnea and an increase in oxygenation index were comparable to those achieved by short-term quitters. Furthermore, patients with mild phlegm, which accounted for 84% of all subjects, showed greater improvement in phlegm in AECOPD patients with long-term cessation of smoking.

**Conclusion:**

It was found that in patients with AECOPD who quit smoking for a long period of time, there was a reduction in symptoms, improvement in lung function, reduction in treatment intensity, and better improvement in phlegm symptoms after therapy. It is beneficial and necessary to quit smoking early, even if you smoke a small amount of cigarettes.

**Supplementary information:**

The online version contains supplementary material available at 10.1186/s12890-022-02187-5.

## Introduction

Chronic obstructive pulmonary disease (COPD) is one of the most prevalent chronic respiratory diseases, characterized by persistent restrictions in airflow and respiratory symptoms. It is the third leading cause of death worldwide, with China accounting for nearly one-third of those deaths [1–3].

Smoking has generally been considered to be the most significant risk factor for COPD [1]. Nearly two-thirds of COPD patients are current or former smokers [4], making it crucial to study their clinical characteristics. A number of studies have demonstrated that continuous smoking adversely impacts symptom control, lung function maintenance, exacerbation frequency, and mortality [5–8], as well as a quantitative relationship between cigarette smoking and ventilatory function [9]. However, only Scanlon explored the effect of different smoking cessation periods on lung function improvement [6] and no study has investigated the impact of different smoking cessation periods on the clinical characteristics and treatment response of COPD patients, particularly those with exacerbated disease.

The purpose of this study is to determine whether there are any differences in clinical characteristics and therapeutic responses between AECOPD patients with different smoking cessation periods. The study recruited current or former smokers with AECOPD. The symptoms, objective indicators, and therapeutic details of short-term and long-term quitters were recorded and compared. COPD patients may benefit from quitting smoking early by not only preventing the harmful effects of cigarettes, but also allowing them to recover from the more severe illness.

## Methods

### Study population

Similar with our previous study [10], from January 2021 until December 2021, the Haian Hospital affiliated with Nantong University screened and employed adults with previously clinically diagnosed COPD and acute exacerbations of symptoms, and at the affiliated Ruijin Hospital of Shanghai Jiao Tong University School of Medicine. As approved by the Institutional Review Boards of affiliated Haian Hospital of Nantong University and Shanghai Jiao Tong University School of Medicine affiliated Ruijin Hospital, the present study followed the Declaration of Helsinki. The present study was conducted with the written informed consent of all adult participants.

Criteria include: (1) Patients clinically diagnosed with AECOPD upon admission; (2) Lung function ascertained prior to the present exacerbation when the patients were in stable condition, with a post-bronchodilator ratio of forced expiratory volume in 1 s/forced vital capacity (FEV1/FVC) less than 70%, conforming to diagnosis criteria of the Global Initiative for COPD (GOLD) [1]; (3) current or former smokers.

Exclusion criteria: Patients suffering from pneumonia, acute asthma exacerbations, active tuberculosis, bronchiectasis, bronchiolitis obliterans, generalized bronchiolitis, or unstable cardiac conditions in the last four months. In addition, patients with a previous clinical diagnosis of asthma or a positive bronchodilator reversibility test prior to the present exacerbation were excluded from the study.

In accordance with previous research on the definition of successful smoking cessation, five years is considered sufficient time to qualify [11, 12]. A threshold of five pack-year was used to distinguish between long-term and short-term smoking cessation because most study participants smoked a pack of cigarettes a day.

### Data collection

During the recruitment process, all patients underwent a medical interview, a physical examination, and laboratory tests. A day-time cough symptom scoring system [8], grading cough symptoms from 0 to 3, with a score of 0 denoting no cough, was used to assess cough severity. Similar to cough severity, phlegm severity was recorded. A modified Medical Research Council scale (mMRC) was used to assess the severity of dyspnea. The symptoms were reassessed 24 h before discharge from the hospital. Critical information about symptoms and smoking status were double checked by two different researchers and discrepancies were addressed by discussion among all the researchers.

Spirometry was performed in accordance with the recommendations of the American Thoracic Society (ATS) and European Respiratory Society (ERS) when patients were stable [9]. We assessed FEV1, FEV1% of predicted value (FEV1%), FEV1/FVC, the ratio of residual volume to total lung capacity (RV/TLC), the diffusing capacity of the lung for carbon monoxide (DLCO), and the ratio of DLCO to alveolar volume (DLCO/VA).

An arterial blood gas analysis was performed upon admission and 24 h prior to discharge to determine the arterial oxygenation index (calculated as oxygen partial pressure (mmHg) divided by oxygen fraction inspired) and carbon dioxide partial pressure (PaCO_2_).

The use of medications (e.g., antibiotics, inhaled corticosteroid (ICS), long-acting β2 agonists (LABA) or long-acting anti-muscarinic antagonists (LAMA), short-acting β2 agonists (SABA), and systemic corticosteroid) was recorded. Additionally, the non-invasive ventilation time and the hospital stay were recorded.

### Statistical analysis

Numbers (percentages) are used for categorical variables, while the mean ± standard deviations (SD) are used for continuous variables. In order to compare differences between the groups, Fisher’s exact test was used for categorical variables, and one-way analysis of variance (ANOVA) or non-parametric Mann-Whitney tests were used for continuous or ordinal variables, respectively. Analysis of covariance and logistic regression analyses were conducted taking into account possible confounding factors.

Statistical analyses were performed using IBM SPSS, version 20.0 (IBM Corp., Armonk, NY, USA), and a p-value of < 0.05 was considered significant.

## Results

1. **Demographic characteristics of AECOPD patients who have ceased smoking at different times.**

**Fig. 1:**
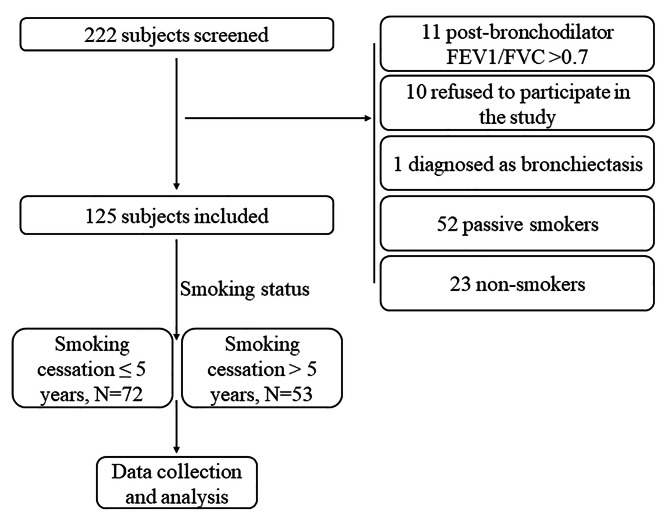
Flowchart of the study

The study screened 222 patients, and ultimately 125 patients were covered (see Fig. 1). Seventy-two of these patients had quit smoking no more than five years ago, three were current smokers who had quit smoking during this period of disease exacerbation, and 53 had quit smoking more than five years ago. The subjects were all male. A balance was observed across the groups in terms of characteristics such as age, BMI, educational level, occupation, occupation exposure, biomass exposure, and prior respiratory disease. The short-term quitters smoked more (smoking pack-year, 11.3 ± 3.8 vs. 9.8 ± 2.4, *P* < 0.01, short-term and long-term quitters, respectively), but this difference was not observed among smokers who did not exceed 10 pack-years (8.4 ± 2.0 vs. 8.8 ± 1.6, *P* > 0.05). A more detailed description of demographic characteristics can be found in Table 1.

**Table 1:** Demographic Characteristic of subjects

	COPD quitting smoking ≤ 5 years (N = 72)	COPD quitting smoking > 5 years (N = 53)	*P* value
Sex, N (%)			**/**
Male	72 (100.0)	53 (100.0)	
Female	0 (0.0)	0 (0.0)	
Age, year	66.1 ± 8.1	66.91 ± 8.8	0.616
BMI, kg/m^2^	22.6 ± 3.3	23.0 ± 3.5	0.444
Education, N (%)			
Illiteracy	9 (12.5)	13 (24.5)	0.374
Primary school graduate	27 (37.5)	16 (30.2)	
Junior high school graduate	27 (37.5)	18 (34.0)	
Senior high school graduate	9 (12.5)	6 (11.3)	
Occupation, N (%)			0.685
Worker	14 (19.4)	13 (24.5)	
Farmer	36 (50.0)	21 (39.6)	
Retirement	21 (29.2)	18 (34.0)	
Self-employed	1 (1.4)	1 (1.9)	
Smoking amount, pack-year	11.3 ± 3.8	9.8 ± 2.4	**0.008**
Smoking Cessation Time, y	3.7 ± 1.4	9.3 ± 3.1	**0.000**
Occupational exposure, N (%)	4 (5.6)	5 (9.4)	0.493
Occupational exposure time, year	0.8 ± 4.6	2.7 ± 8.6	0.142
Biomass exposure, N (%)	13 (18.1)	9 (17.0)	1.000
Biomass exposure time, year	6.7 ± 14.7	6.5 ± 14.6	0.938
Prior respiratory diseases, N (%)	5 (6.9)	3 (5.7)	1.000

Data were represented as mean ± standard deviation (SD) except for particular specifications. BMI, body mass index

2. **In AECOPD patients with a longer smoking cessation time, the severity of the disease and the number of acute exacerbations were less severe.**

**Fig. 2:**
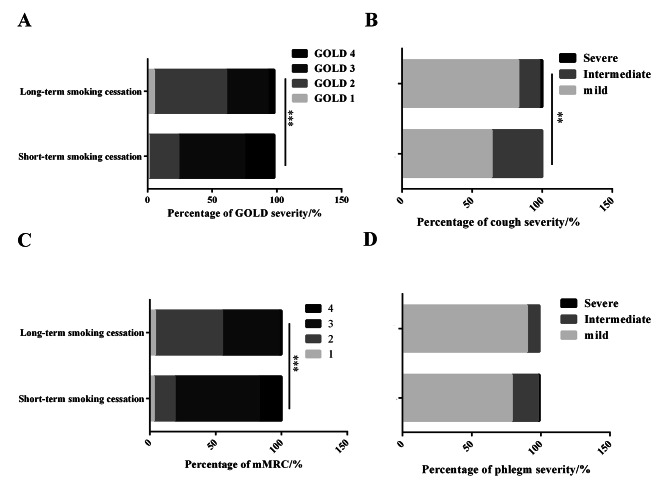
Proportion of patients with different (A) GOLD grade classification, (B) cough severity, (C) mMRC score, and (D) phlegm severity. **P < 0.01, and ***P < 0.001

It is important to note that although there was no difference between short-term and long-term quitters in the proportion of group D patients (93.1 vs. 84.9%, *P* > 0.05, according to the ABCD assessment tool of GOLD guideline), patients who had longer periods without smoking were more likely to experience mild airway obstruction (percentage of patients with GOLD grade severity 1 or 2, 25.0 vs. 62.3%, *P* < 0.001, short-term and long-term quitters, respectively, Fig. 2 A**)**. Additionally, in spite of the fact that long-term smoking cessation did not influence phlegm severity (percentage of patients with mild phlegm, 79.2 vs. 90.6%, *P* > 0.05), patients with long-term smoking cessation reported milder dyspnea and cough symptoms (percentage of patients with mild cough, 63.9 vs. 83.0%, *P* < 0.05, Fig. 2B; percentage of patients with mMRC score < 3, 19.4 vs. 54.7%, *P* < 0.001, Fig. 2 C).

In terms of objective indicators, smoking cessation for a longer period of time was associated with a higher oxygenation index (222.5 ± 42.1 vs. 253.1 ± 36.8mmHg, *P* < 0.001), lower PaCO_2_ (73.7 ± 11.8 vs. 61.5 ± 10.3mmHg, *P* < 0.001), and a milder form of pulmonary hypertension, airflow restriction, gas retention, and acute exacerbations (systolic pulmonary arterial pressure (SPAP), 50.0 ± 9.5 vs. 42.3 ± 8.4mmHg, *P* < 0.001; FEV1, 1.1 ± 0.4 vs. 1.6 ± 0.6 L, *P* < 0.001; FEV1%, 40.7 ± 16.1 vs. 57.5 ± 16.8%, *P* < 0.001; RV/TLC, 57.0 ± 10.9 vs. 51.7 ± 7.8%, *P* < 0.01; number of acute exacerbations per year, 2.7 ± 1.0 vs. 1.9 ± 1.0, *P* < 0.001). In accordance with this phenomenon, AECOPD patients who had stopped smoking for a longer period of time were treated less intensively. There was a shorter course of antibiotics (9.6 ± 1.7 vs. 8.7 ± 1.1 days, *P* < 0.01), a reduced use of systemic glucocorticoids (137.8 ± 67.8 vs. 100.0 ± 78.8 mg, *P* < 0.01), and a shorter duration of non-invasive ventilation (6.8 ± 4.5 vs. 1.9 ± 3.8 days, *P* < 0.001, Table 2).

**Table 2:** Clinical and pulmonary functional characteristic of subjects

	COPD quitting smoking ≤ 5 years (N = 72)	COPD quitting smoking > 5 years (N = 53)	*P* value
Oxygenation index, mmHg	222.5 ± 42.1	253.1 ± 36.8	**0.000**
PaCO_2_, mmHg	73.7 ± 11.8	61.5 ± 10.3	**0.000**
SPAP, mmHg	50.0 ± 9.5	42.3 ± 8.4	**0.000**
FEV1, L	1.1 ± 0.4	1.6 ± 0.6	**0.000**
FEV1%	40.7 ± 16.1	57.5 ± 16.8	**0.000**
RV/TLC, %	57.0 ± 10.9	51.7 ± 7.8	**0.003**
DLCO, mmol/min/Kpa	6.1 ± 2.2	6.8 ± 1.7	0.105
DLCO/VA, mmol/min/Kpa/L	1.1 ± 0.3	1.2 ± 0.2	0.148
AE number per year	2.7 ± 1.0	1.9 ± 1.0	**0.000**
Antibiotic time, day	9.6 ± 1.7	8.7 ± 1.1	**0.002**
ICS + LABA use, N (%)	61 (84.7)	34 (64.2)	**0.011**
SABA use, N (%)	30 (41.7)	19 (35.8)	0.580
SABA time, day	4.1 ± 5.0	3.3 ± 4.5	0.318
Dose of systemic glucocorticoids, mg, equivalent dose of methylprednisolone	137.8 ± 67.8	100.0 ± 78.8	**0.006**
NIV time, day	6.8 ± 4.5	1.9 ± 3.8	**0.000**

Data were represented as mean ± SD except for particular specifications. SPAP, systolic pulmonary arterial pressure; FEV1, forced expiratory volume in 1 s; FEV1%, FEV1% of predicted value; RV, residual volume; TLC, total lung capacity; DLCO, diffusing capacity of the lung for carbon monoxide; VA, alveolar volume; AE, acute exacerbations; ICS, inhaled corticosteroid; LABA, long-acting β2 agonists; NIV, non-invasive ventilation. LAMA (long-acting anti-muscarinic antagonist) was used for every patient and data was not shown in the table

As noted above, patients who quitted smoking earlier also smoked less, which affected the severity of COPD as well, according to previous studies [9, 13]. Analysis of covariance or logistic regression was conducted in order to eliminate this possible confounding factor. Similar results were obtained (cough severity, short-term vs. long-term quitters, odds ratio (OR) = 2.0, 95% confidence interval (CI) 0.9 ~ 4.2, *P* > 0.05; mMRC score, OR = 2.5, 95% CI 1.3 ~ 4.8, *P* < 0.01; phlegm severity, OR = 1.5, 95% CI 0.7 ~ 3.5, *P* > 0.05; adjustable oxygenation index, 220.6 ± 39.0 vs. 255.7 ± 39.1mmHg, *P* < 0.001; adjustable PaCO_2_, 73.5 ± 11.3 vs. 61.7 ± 11.3mmHg, *P* < 0.001; adjusted SPAP 50.0 ± 9.1 vs. 42.2 ± 9.1mmHg, *P* < 0.001; adjusted FEV1 1.1 ± 0.5 vs. 1.6 ± 0.5 L, *P* < 0.001; adjusted FEV1%, 41.6 ± 15.9 vs. 56.4 ± 15.9%, *P* < 0.001; adjusted RV/TLC 57.2 ± 9.7 vs. 51.5 ± 9.8%, *P* < 0.01; adjusted number of acute exacerbations per year, 2.7 ± 1.0 vs. 2.0 ± 1.0, *P* < 0.01). Additionally, since short-term and long-term quitters shared similar smoking amounts for patients with ≤ 10 pack-years, these subjects were subjected to a focused analysis to confirm the results. There were 39 short-term quitters and 42 long-term quitters. Similarly, longer smoking cessation was associated with milder dyspnea, cough, and phlegm symptoms (percentage of patients with mMRC score < 3, 23.1 vs. 57.1%, *P* < 0.01; percentage of patients with mild cough, 61.5 vs. 88.1%, *P* < 0.01; percentage of patients with mild phlegm, 76.9 vs. 92.9%, *P* < 0.05). In addition, there was a higher oxygenation index (211.2 ± 47.7 vs. 254.3 ± 36.1mmHg, *P* < 0.001), a lower PaCO_2_ (73.0 ± 13.0 vs. 60.9 ± 9.7mmHg, *P* < 0.001), and a milder form of pulmonary hypertension, airflow restriction, gas retention, and acute exacerbations in long-term quitters (SPAP, 50.0 ± 10.0 vs. 41.6 ± 8.3mmHg, *P* < 0.001; FEV1, 1.2 ± 0.5 vs. 1.7 ± 0.6 L, *P* < 0.001; FEV1%, 42.9 ± 16.8 vs. 59.5 ± 16.6%, *P* < 0.001; RV/TLC, 58.6 ± 12.0 vs. 51.5 ± 8.1%, *P* < 0.01; number of acute exacerbations per year, 2.6 ± 1.0 vs. 1.9 ± 0.9, *P* < 0.01).

3. **Patients with long-term smoking cessation with AECOPD showed more obvious improvements in phlegm.**

Despite the fact that patients with short-term smoking cessation received more intensive treatment, their therapeutic response was similar to that of patients with long-term smoking cessation, except for coughing symptoms and PaCO_2_ improvement. In AECOPD patients with short-term smoking cessation (Table 3), there was an better improvement in cough symptoms (37.5 vs. 18.9%, *P* < 0.05, short-term and long-term quitters, respectively) and PaCO_2_ (-14.4 ± 8.0 vs. -6.4 ± 6.2mmHg, *P* < 0.001), which may be due to more severe cough symptoms (correlation coefficient 0.960, *P* < 0.001) and CO_2_ retention (correlation coefficient − 0.939, *P* < 0.001). For patients with mild phlegm, who accounted for 84% of all the subjects, the improvement in phlegm was more pronounced in AECOPD patients with long-term smoking cessation (percentage of patients with phlegm improvement, 71.9 vs. 89.6%, *P* < 0.05).

**Table 3:** Therapeutic response of AECOPD patients with different smoking cessation time

	COPD quitting smoking ≤ 5 years (N = 72)	COPD quitting smoking > 5 years (N = 53)	*P* value
Subjects with mMRC improvement, N (%)	34 (47.2)	18 (34.0)	0.147
Subjects with cough improvement, N (%)	27 (37.5)	10 (18.9)	**0.030**
Subjects with expectoration improvement, N (%)	56 (77.8)	48 (90.6)	0.089
Subjects with expectoration improvement for mild expectoration, N (%)	41 (71.9)	43 (89.6)	**0.029**
Oxygenation index improvement, mmHg	34.2 ± 14.1	29.9 ± 20.2	0.166
PaCO_2_ improvement, mmHg	-14.4 ± 8.0	-6.4 ± 6.2	**0.000**
Hospital time, day	9.5 ± 1.4	8.7 ± 1.2	**0.003**

Data were represented as mean ± SD except for particular specifications

## Discussion

Cigarette smoking, especially active smoking, has long been considered one of the most significant risk factors for COPD, and smoking cessation is recognized as one of the most effective ways to slow the progression of the disease. Although previous studies have mainly focused on the negative effects of continuous smoking on COPD patients, few studies have elaborated on the positive effects of smoking cessation on COPD patients. In our study, the majority of patients (97.6%) had quitted smoking for more than a year, allowing us to evaluate the impact of smoking cessation duration on the clinical characteristics of AECOPD patients. As expected, The effects of long-term cessation of smoking on AECOPD patients were more positive than those of short-term cessation, including reduced symptoms, improved pulmonary function, less intense therapy, and better improved phlegm after treatment.

There has been a correlation between impaired lung function and cigarette smoking since the 1960s, and Burrows’ study also established a quantitative relationship between smoking and reduced lung function [9]. Moreover, several studies have demonstrated that continuous smoking can result in persistent symptoms, reduced lung function, frequent exacerbations, and high mortality rates among COPD patients [6, 8, 14, 15]. According to our previous study, non-smokers with AECOPD had less severe disease symptoms than those who smoked [10]. However, only Paul’s study examined the effect of smoking cessation time duration and found that those who stopped smoking experienced improvements in FEV1 one year after quitting, and the subsequent decline in FEV1 was comparable with that of non-smokers [6]. This suggests that COPD patients can recover from obstructive airway limitation to some extent after smoking cessation. This conclusion was supported by our findings. In addition, long-term smoking cessation significantly reduced cough, dyspnea, hypoxemia, CO_2_ retention, and pulmonary arterial hypertension, suggesting that long-term smoking cessation has significant and extensive benefits.

We found that patients who had stopped smoking for a longer time were also treated less intensively, similar to our previous study, which found that non-smokers were also treated less intensively [10]. The treatment response was not significantly different between patients with different cessation times, except for improvement in cough, phlegm, and CO_2_ retention. There was a greater improvement in cough symptoms and CO_2_ retention in patients with short-term smoking cessation, potentially due to more severe baseline disease conditions and more intensive treatment strategies, especially non-invasive ventilation, and a decreased chance that mild cough would improve after treatment (percentage of improvement, 2% vs. 100% vs. 100%, mild, intermittent, and severe cough, respectively). Phlegm improvement was more pronounced in AECOPD patients who had stopped smoking for a longer period of time, possibly due to improved mucociliary function and decreased goblet cell hyperplasia as a result of cessation of smoking [16].

The present study was not without limitations. First, as an observational study, even with multiple statistical methods, it is not possible to balance all confounding factors between groups. Besides, selection and patient recall bias could not be completely avoided even we did our best to deal with them by recruiting patients from hospitals located in urban (Shanghai) and suburban (Haian) areas, screening all the possible AECOPD patients admitted to the hospitals, double checking critical medical history information by two different researchers. In addition, the number of subjects was limited, making it difficult to extrapolate the results to a larger population. Finally, the findings of this study may not apply to patients with mild AECOPD, as it included patients with severe AECOPD requiring hospitalization.

## Conclusion

A unique perspective was presented in this study regarding the impact of smoking cessation on AECOPD patients. Furthermore, different smoking cessation time durations led to different clinical characteristics and treatment responses in patients with AECOPD. Symptoms, hypoxemia, CO_2_ retention, and lung function impairment were less severe in patients who had stopped smoking for a long period of time as opposed to those who had stopped smoking for a short period of time. In addition, patients who had stopped smoking for a long period of time had a better improvement in phlegm, even with a lower intensity of treatment. Early smoking cessation not only prevents the harms caused by smoking, but also improves patients’ health. Even for patients with small amounts of smoking, early cessation was beneficial and necessary.

## Electronic supplementary material

Below is the link to the electronic supplementary material.

Supplementary Material 1

Supplementary Material 2

## Data Availability

Upon reasonable request, the corresponding author will provide the datasets used and/or analyzed during the current study.

## List of abbreviations

COPD: chronic obstructive pulmonary disease
AECOPD: acute exacerbation of COPD
mMRC: modified Medical Research Council scale
FEV1: forced expiratory volume in 1 s
FVC: forced vital capacity
GOLD: Global Initiative for COPD
ATS: American Thoracic Society
ERS: European Respiratory Society
FEV1%: FEV1% of predicted value
RV: residual volume
TLC: total lung capacity
DLCO: diffusing capacity of the lung for carbon monoxide
VA: alveolar volume
PaCO_2_: carbon dioxide partial pressure
ICS: inhaled corticosteroid
LABA: long-acting β2 agonists
LAMA: long-acting anti-muscarinic antagonists
SABA: short-acting β2 agonists
SD: standard deviation
ANOVA: analysis of variance
BMI: body mass index
SPAP: systolic pulmonary arterial pressure
OR: odds ratio
CI: confidence interval
