# Supplementary material for: An observational study of the effects of smoking cessation earlier on the clinical characteristics and course of acute exacerbations of chronic obstructive pulmonary disease
Source: BMC Pulm Med. 2022 Oct 27;22:390. doi: 10.1186/s12890-022-02187-5 (PMC9615224; doi:10.1186/s12890-022-02187-5)
Supplement: Supplementary file 2 — Supplementary Material 2 [file 12890_2022_2187_MOESM2_ESM.docx]

**Table S1**. Demographic Characteristic of subjects.

|  | COPD quitting smoking ≤ 5 years (N=69) | COPD quitting smoking > 5 years (N=53) | *P* value |
| --- | --- | --- | --- |
| Sex, N (%) |  |  | **/** |
| Male | 69 (100.0) | 53 (100.0) |  |
| Female | 0 (0.0) | 0 (0.0) |  |
| Age, year | 66.6±7.9 | 66.91±8.8 | 0.868 |
| BMI, kg/m^2^ | 22.4±3.2 | 23.0±3.5 | 0.314 |
| Education, N (%) |  |  |  |
| Illiteracy | 9 (13.0) | 13 (24.5) | 0.416 |
| Primary school graduate | 27 (39.1) | 16 (30.2) |  |
| Junior high school graduate | 25 (36.2) | 18 (34.0) |  |
| Senior high school graduate | 8 (11.6) | 6 (11.3) |  |
| Occupation, N (%) |  |  | 0.334 |
| Worker | 12 (17.4) | 13 (24.5) |  |
| Farmer | 36 (52.2) | 21 (39.6) |  |
| Retirement | 21 (30.4) | 18 (34.0) |  |
| Self-employed | 0 (0.0) | 1 (1.9) |  |
| Smoking amount, pack-year | 11.2±3.9 | 9.8±2.4 | **0.012** |
| Smoking Cessation Time, y | 3.9±1.2 | 9.3±3.1 | **0.000** |
| Occupational exposure, N (%) | 3 (4.3) | 5 (9.4) | 0.292 |
| Occupational exposure time, year | 0.8±4.7 | 2.7±8.6 | 0.152 |
| Biomass exposure, N (%) | 13 (18.8) | 9 (17.0) | 0.817 |
| Biomass exposure time, year | 7.0±14.9 | 6.5±14.6 | 0.854 |
| Previous pulmonary diseases, N (%) | 5 (7.2) | 3 (5.7) | 1.000 |

Data were represented as mean ± standard deviation (SD) except for particular specifications. BMI, body mass index.

**Table S2**. Clinical and pulmonary functional characteristic of subjects.

|  | COPD quitting smoking ≤ 5 years (N=69) | COPD quitting smoking > 5 years (N=53) | *P* value |
| --- | --- | --- | --- |
| Oxygenation index, mmHg | 219.6±40.5 | 253.1±36.8 | **0.000** |
| PaCO_2_, mmHg | 74.7±11.0 | 61.5±10.3 | **0.000** |
| SPAP, mmHg | 50.7±8.8 | 42.3±8.4 | **0.000** |
| FEV1, L | 1.1±0.4 | 1.6±0.6 | **0.000** |
| FEV1% | 39.3±14.5 | 57.5±16.8 | **0.000** |
| RV/TLC, % | 57.4±11.0 | 51.7±7.8 | **0.002** |
| DLCO, mmol/min/Kpa | 6.1±2.2 | 6.8±1.7 | 0.084 |
| DLCO/VA, mmol/min/Kpa/L | 1.1±0.3 | 1.2±0.2 | 0.122 |
| Antibiotic time, day | 9.7±1.7 | 8.7±1.1 | **0.000** |
| ICS+LABA use, N (%) | 61 (88.4) | 34 (64.2) | **0.002** |
| SABA use, N (%) | 30 (43.5) | 19 (35.8) | 0.458 |
| SABA time, day | 4.3±5.0 | 3.3±4.5 | 0.233 |
| Dose of systemic glucocorticoids, mg, equivalent dose of methylprednisolone | 143.8±62.6 | 100.0±78.8 | **0.001** |
| NIV time, day | 7.1±4.4 | 1.9±3.8 | **0.000** |

Data were represented as mean ± SD except for particular specifications. SPAP, systolic pulmonary arterial pressure; FEV1, forced expiratory volume in 1 second; FEV1%, FEV1 percentage of predicted value; RV, residual volume; TLC, total lung capacity; DLCO, diffusing capacity of the lung for carbon monoxide; VA, alveolar volume; ICS, inhaled corticosteroid; LABA, long-acting β2 agonists; NIV, non-invasive ventilation.

**Table S3**. Therapeutic response of AECOPD patients with different smoking cessation time.

|  | COPD quitting smoking ≤ 5 years (N=69) | COPD quitting smoking > 5 years (N=53) | *P* value |
| --- | --- | --- | --- |
| Subjects with mMRC improvement, N (%) | 34 (49.3) | 18 (34.0) | 0.100 |
| Subjects with cough improvement, N (%) | 27 (39.1) | 10 (18.9) | **0.018** |
| Subjects with expectoration improvement, N (%) | 53 (76.8) | 48 (90.6) | 0.055 |
| Subjects with expectoration improvement for mild expectoration, N (%) | 38 (70.4) | 43 (89.6) | **0.026** |
| Oxygenation index improvement, mmHg | 35.0±13.9 | 29.9±20.2 | 0.103 |
| PaCO_2_ improvement, mmHg | -15.0±7.7 | -6.4±6.2 | **0.000** |
| Hospital time, day | 9.6±1.3 | 8.7±1.2 | **0.001** |

Data were represented as mean ± SD except for particular specifications.
